# Remission of HIV-related naïve and high-risk Burkitt’s lymphoma treated by autologous stem cell transplantation plus cART

**DOI:** 10.1186/s13287-018-1089-5

**Published:** 2018-12-20

**Authors:** Haiyan Min, Jianwei Yang, Sanbin Wang, Pengfei Tao, Yuqin Song, Xiaopei Wang, Huiqin Li, Xinping Yang, Xingqi Dong, Fu-Sheng Wang, Ming Shi, Xicheng Wang, Ruonan Xu

**Affiliations:** 1 Yunnan Provincial Hospital of Infectious Diseases, Kunming, 650301 China; 2 The Third People’s Hospital of Yunnan Province, Kunming, 650011 China; 3 Kunming General Hospital of Chengdu Military Region, Kunming, 650118 China; 4 Beijing Cancer Hospital, Peking University, Beijing, 100142 China; 5 Treatment and Research Centre for Infectious Disease, Beijing 302 Hospital, Beijing, 100039 China

**Keywords:** HIV, Naïve, Burkitt’s lymphoma, Autologous stem cell transplantation, Antiretroviral therapy, CD4+T

## Abstract

A 27-year-old male with HIV-associated naïve and high-risk Burkitt’s lymphoma sequentially received short-term, high-dose non-myeloablative chemotherapy and autologous CD34-positive stem cell transfusion in the setting of combined antiretroviral therapy (cART). Prompt hematopoietic recovery was observed after 2 weeks and clinical remission from Burkitt’s lymphoma within approximately 30 months after transplantation. The HIV RNA load was inhibited persistently, and drug resistance was not observed. The CD4+ T cell count approached 323 cells/μL in a recent follow-up study. This case suggests that the use of intensive non-myeloablative chemotherapy with transplantation, combined with antiretroviral therapy, in HIV-related naive and high-risk Burkitt’s lymphoma was tolerated and safe.

## Background

Antiretroviral therapy (ART) has changed the outcome of patients with human immunodeficiency virus (HIV) infection by efficiently inhibiting virus replication, recovering the cluster of CD4+ T cell count, and reducing the risk of opportunistic infections [1]. However, HIV-associated adverse events will continue to occur in some patients despite ART, especially in those patients who never achieve complete immunological reconstitution.

Burkitt’s lymphoma (BL) accounts for < 2% of the total cases of lymphoma in China. BL originates from a germinal center that is closely related to *c-Myc* rearrangement and infection with the Epstein–Barr virus (EBV). More than half of patients with BL have advanced disease when diagnosed [2]. A combination of ART with intensive chemotherapy can lead to good outcomes for BL. Furthermore, patients with HIV-associated lymphomas (HRLs) have been considered candidates for allogeneic or autologous stem cell transfusion (ASCT) when the criteria for transplantation have been met [3]. Currently, patients with relapsed/persistent HRLs have achieved good outcomes, comparable with those of non-HIV-infected individuals, after transfusion of stem cells [4]. In the present study, we report the outcome for a patient with HIV-associated naïve and high-risk BL who received ASCT after intensive chemotherapy plus combined antiretroviral therapy (cART).

## Materials and methods

### Patient

A 27-year-old male was admitted to our unit upon presentation of a painless mass in the right groin in April 2015. Biopsies of the lesion revealed lymphoma, and antibodies against HIV were positive. He refused to accept any treatment for the concomitant HIV infection. In the following 3 months, the mass became larger and ulcers formed on the skin. In addition, the right thigh also became involved. He suffered from recurrent fever, with a body temperature fluctuating from 38 to 40.5 °C, and his body weight decreased by approximately 12 kg within 3 months.

In August 2015, a biopsy of the mass aspirate showed BL, and the immunohistochemical results were positive for CD20 and EBV-encoded RNA (EBER)1/2. In addition, a bone marrow biopsy showed the total chromosomes to be normal, whereas the percentage of unidentified cells was 1.8%.

Using positron emission tomography–computed tomography (PET–CT), we found increased abnormal metabolism of fludeoxyglucose (FDG) in the right groin; the region had dimensions (in cm) of 12.0 × 16.5 × 27.0, and the boundaries were not clear. The right thigh, anterior to the bilateral mandible, neck, axillary, retroperitoneal vessel, right iliac fossa, pelvic wall, and right inguinal lymph nodes shown increased metabolism of FDG. A blood count showed abnormal levels of lactate dehydrogenase (LDH; 1579 U/L) as well as a white blood cell (WBC) count of 4.42 × 10^9^/L, a neutrophil count of 2.92 × 10^9^/L, a hemoglobin level of 122 g/L, and platelet count of 330 × 10^9^/L. The patient was diagnosed as having stage IV BL.

The HIV RNA load was 51,386 copies/mL, and the CD4+ T cell count was 107 cells/μL at the time of BL diagnosis. In addition, the patient was co-infected with EBV, and the EBV DNA load was 4.09 × 10^4^ copies/mL. No other serious opportunistic infections were present, and the CD4+ T cell count was < 200 cells/μL; therefore, he was started on cART immediately with lamivudine (300 mg daily), tenofovir (300 mg daily), and efavirenz (600 mg daily) on the 25th of August 2015. These medications were not changed, and drug resistance did not occur.

The patient was started on a standard dose of rituximab, piraubicin, vincristine, etoposide, cyclophosphamide, and prednisone acetate (R-EPOCH) chemotherapy on the 31st of August 2015 (day 0). The patient developed a fever on the 6th of September because of a *Staphylococcus aureus* infection in his ulcer, based on a drug-sensitivity test. The patient is allergic to penicillin; therefore, he received lincomycin (400 mg, q.d.s.). Local debridement was performed every day, and his temperature returned to normal 7 days after treatment. Lincomycin was stopped on day 16 because of a continuous negative result in blood culture. He was diagnosed with agranulemia (neutrophil count = 0.01 × 10^9^/L) on day 13, and granulocyte-colony stimulating factor (G-CSF; 150 μg, q.d.s) was given until the neutrophil count reached 0.5 × 10^9^/L. Meanwhile, he suffered a pulmonary infection and meropenem (0.5 g, b.d.s.) was added until the symptoms were controlled completely (achieved on day 30). A bone marrow biopsy showed that proliferation of the bone marrow was active, and abnormal cells were not detected.

The second cycle of chemotherapy comprised rituximab, methotrexate, and cytosine arabinoside (Ara-C). It was started on the 21st of September (day 22) and stopped on the 23rd of September (day 24). No serious complications occurred, and the EBV DNA load was reduced to 5 × 10^3^ copies/mL on day 30.

From the 9th of October (day 40) to the 23rd of October (day 54), he received a third cycle of chemotherapy with rituximab, cyclophosphamide, vinorelbine, pirarubicin, and dexamethasone (R + HyperCVAD). The EBV DNA load was undetectable after the third cycle of chemotherapy.

**Fig. 1:**
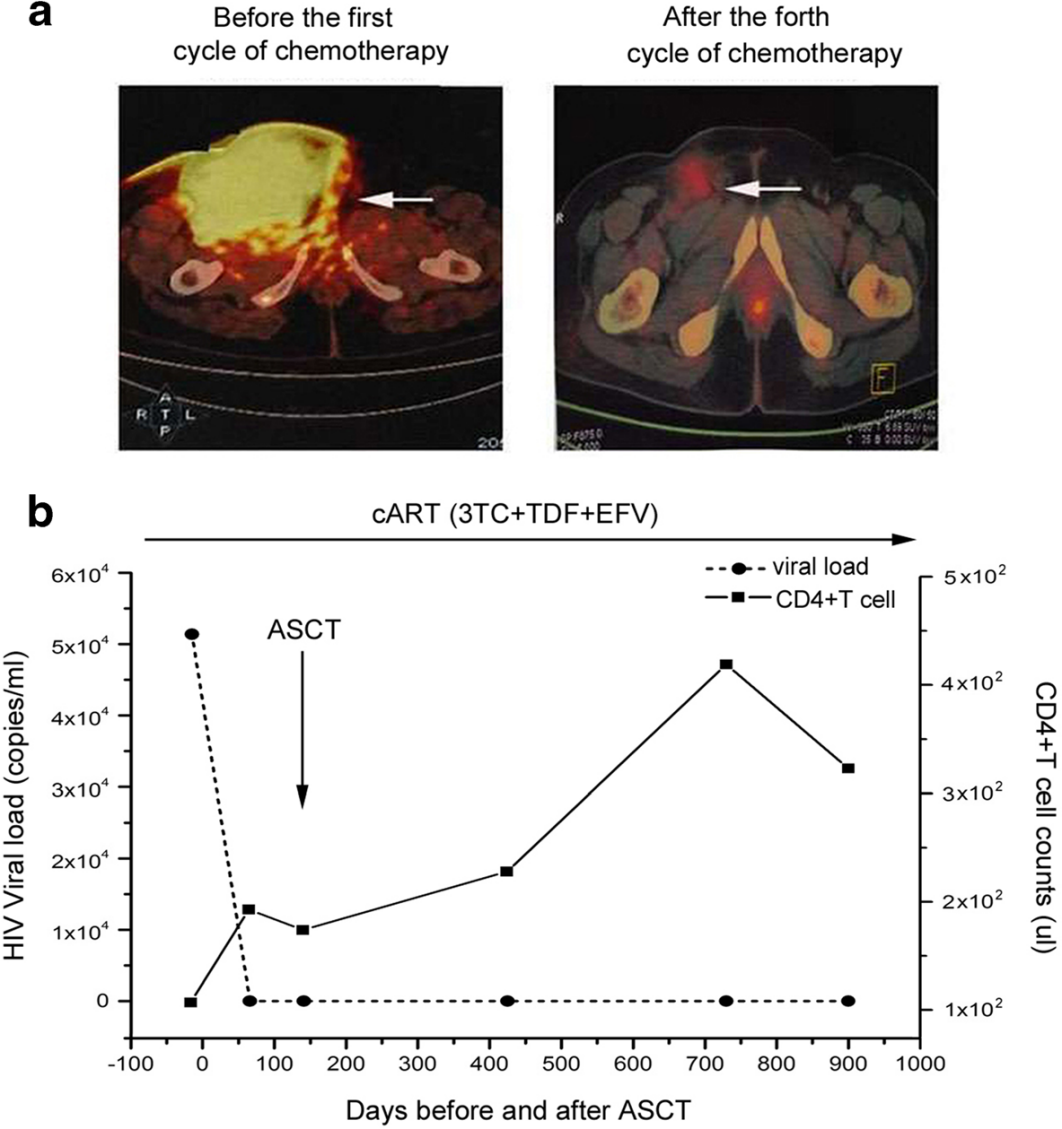
**a** Tumor regression in the right groin after chemotherapy. Tumor evaluation by positron emission tomography–computed tomography is shown before the first cycle and after the fourth cycle of R-EPOCH. **b** HIV RNA load in plasma and CD4+ T cell count before and after autologous bone marrow transplantation. Continuous lines show the viral load and CD4+ T cell counts measured using a standard clinical test before and after autologous stem cell transplantation. 3TC, lamivudine; TDF, tenofovir; EFV, efaviren; R-EPOCH, rituximab, etoposide phosphate, prednisone, vincristine sulfate (Oncovin), cyclophosphamide, doxorubicin hydrochloride (hydroxydaunorubicin)

From the 30th of October (day 61) to the 1st of November (day 63), a fourth cycle of chemotherapy (rituximab plus methotrexate/Ara-C (R-MA)) was undertaken. In addition, six intrathecal injections of methotrexate, Ara-C, and dexamethasone were used during the second and fourth cycles of chemotherapy. After the fourth cycle of chemotherapy, the HIV RNA load was < 40 copies/mL, the CD4+ T cell count was 193 cells/μL, and the LDH level was 91 U/L. PET–CT showed that the tumor volume in the right groin and the maximum standardized uptake value (SUVmax) were reduced significantly, which suggested that tumor activity had been inhibited (Fig. 1a).

**Fig. 2:**
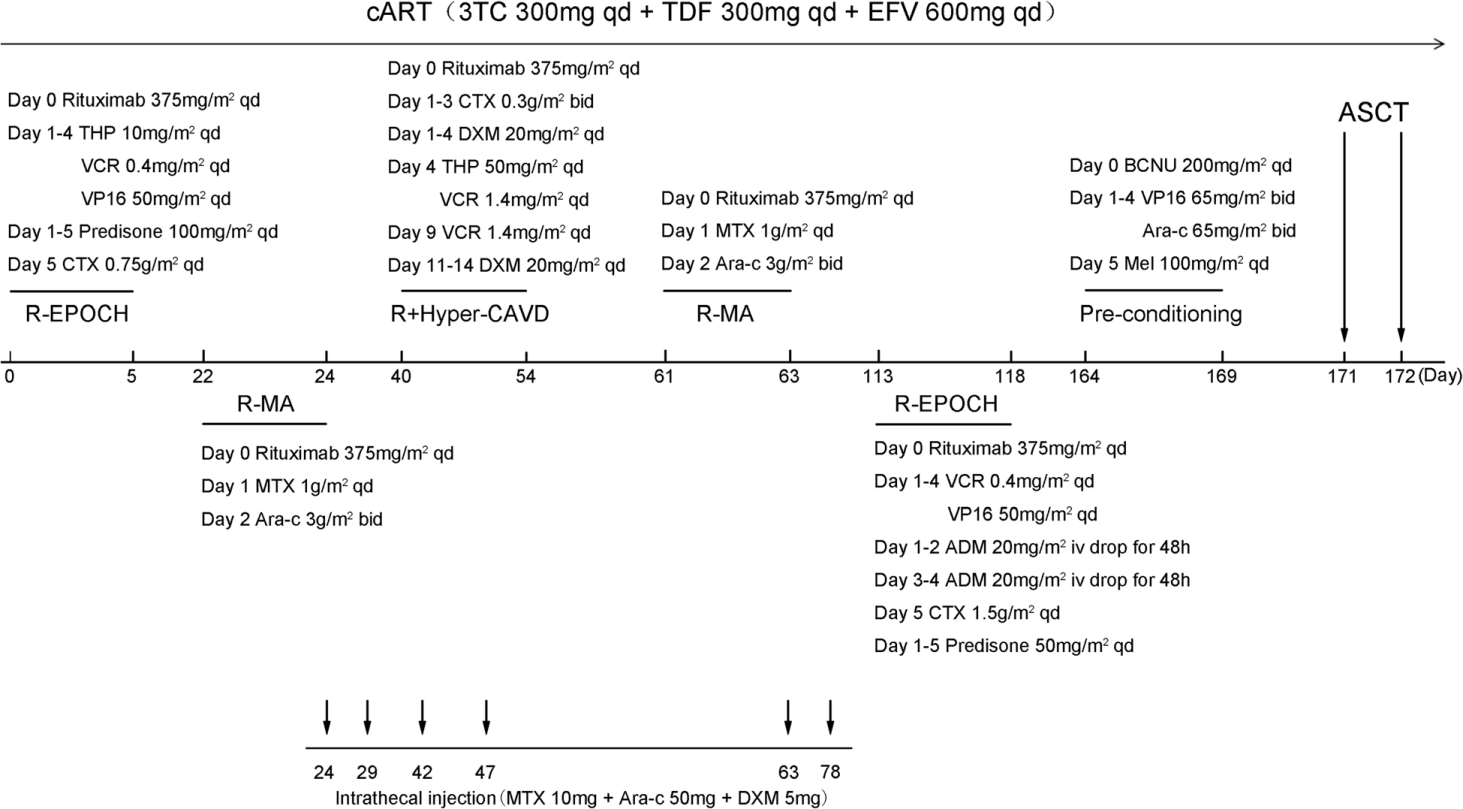
The clinical course and treatment of the patient. cART, combined antiretroviral therapy; THP, piraubicin; VCR, vincristine; VP16, etoposide; CTX, cyclophosphamide; DXM, dexamethasone; MTX, methotrexate; Ara-C, cytosine arabinoside; ADM, pirarubicin; BCNU, carmustine; Mel, melphalan; ASCT, autologous stem cell transfusion

During the second and fourth cycles of chemotherapy, when the neutrophil count was < 0.5 × 10^9^/L, G-CSF (150 μg, q.d.s.) was administered until the neutrophil count reached 0.5 × 10^9^/L. Symptoms of myelosuppression were treated by infusion of packed red blood cells (RBCs) and concentrated platelets, and oral mucositis was treated by gargling with chlorhexidine. The details of the chemotherapy regimens are summarized in Fig. 2.

### Apheresis and processing of peripheral blood stem cells (PBSCs)

Before stem cell mobilization, the R-EPOCH regimen was used between the 20th of November (day 113) and the 25th of November (day 118) 2015 (Fig. 2). On the 27th of November, the WBC count was 0.5 × 10^9^/L, and G-CSF (5 μg/kg per day) was used for PBSC mobilization for the next 5 days. When the WBC count reached 4 × 10^9^/L on the 2nd of December (day 125), PBSC apheresis was undertaken using a continuous blood-cell separator (COBE® Spectra; Terumo BCT, Lakewood, CO, USA). Then, CD34-positive cells were analyzed using a FACScan™ flow cytometer (Becton Dickinson, Franklin Lakes, NJ, USA). A total of 1.03 × 10^6^/kg CD34-positive cells were obtained after undertaking apheresis twice on two consecutive days. PBSC products were combined with a cytoprotectant containing a final concentration of 10% dimethylsulfoxide (Research Industries, Salt Lake City, UT, USA) and freezing at − 150 °C for transfusion.

### Conditioning regimen and PBSC transfusion

The conditioning regimen consisted of carmustine, etoposide, Ara-C, and melphalan, which began on the 11th of January (day 164) and stopped on the 16th of January 2016 (day 169). During PBSC transfusion, only myelosuppression occurred, and serious infections were not observed. Neutropenia was treated by G-CSF (1.5 μg/kg per day), and symptoms of anemia and thrombocytopenia were treated by infusion of packed RBCs and concentrated platelets. Mobilized PBSCs were infused 2 days after completion of melphalan treatment on the 18th of January (day 171) and the 19th of January (day 172).

## Results

### Remission status

After the fourth cycle of chemotherapy, PET–CT scans of the bilateral mandible and neck, chest, abdomen, spine, and big vessels in the para-retroperitoneal, pelvic bones, and bilateral upper femur showed that multiple FDG metabolic abnormalities had disappeared. A follow-up study using bone marrow biopsies showed no morphological evidence of lymphoma, and laboratory studies did not show abnormal blood parameters. The patient remains in complete remission from his lymphoma 30 months after transplantation. In addition, after the third cycle of chemotherapy, EBV DNA was undetectable, and the EBV infection has never recurred in the follow-up study.

### Engraftment

The patient reached a total neutrophil count of 0.5 × 10^9^/L at 14 days and a platelet count > 25 × 10^9^/L without further infusion of platelets at 25 days after ASCT. A bone marrow biopsy showed active proliferation of nucleated cells, as well as recovery of normal hematopoiesis of the bone marrow at 30 days after ASCT.

### HIV viral load and CD4+ T cell count

The patient has remained in complete remission from BL for 30 months after ASCT. The HIV RNA load was < 40 copies/mL after the fourth cycle of chemotherapy, and the CD4+ T cell count was 323 cells/μL in August 2018, as shown in Fig. 1b.

## Discussion

With the development of cART, the clinical outcome and life expectancy of HIV-infected patients have improved. Nevertheless, some patients with a low CD4+ T cell count (< 200 cells/μL) have a greater probability of acquiring HIV-associated and non-HIV-associated events [5]. The risk of developing HIV-related lymphoma in patients with AIDS is much higher than that in patients without AIDS [6].

Although studies have shown that chemotherapy and cART strategies are feasible and tolerated in patients with HIV [7, 8], however, it has been suggested that the use of cART plus chemotherapy should be considered carefully because of the emergence of treatment-related anemia, neurotoxicity, and mucositis; hence, there has been very limited enthusiasm for the use of high-dose chemotherapy plus cART for patients with HIV-associated lymphoma. In our study, cART was started immediately after diagnosis and throughout the course of treatment, viral duplication was well controlled, and viral rebound and drug resistance did not occur. In addition, both the standard chemotherapeutic approaches and chemoimmunotherapy were used and well tolerated.

For patients with HIV-related lymphoma, different doses of chemotherapy have been adopted considering their cytotoxicity and safety. Low and standard doses of chemotherapy carry a similar incidence of adverse effects; however, the prevalence of complete remission is 30% and 48%, respectively [9], which suggests that a standard dose of chemotherapy should be used. Apart from the use of a standard dose of chemotherapy, short-term, high-intensity chemotherapy combined with central prevention has also been reported to improve significantly the survival of HIV patients with high-risk BL [10], which suggests that active chemotherapy may benefit patients with HIV-related lymphoma.

Meanwhile, the availability of rituximab has changed treatment outcomes for patients with BL or B cell lymphoma. Particularly in patients with HIV-associated CD20-positive non-Hodgkin lymphoma, rituximab plus concurrent EPOCH chemotherapy is highly effective [11]. Four cycles of rituximab with EPOCH and hyper-CVAD regimens were used in our chemotherapy regimen, and one cycle of rituximab was used for conditioning. This strategy did not lead to serious treatment-related toxicities during chemotherapy or subsequent ASCT. More importantly, concomitant chemoimmunotherapy with cART was never stopped because of drug interactions. These results suggested that molecular-targeted drugs combined with cART were safe and well tolerated.

Sequential therapy combined with ASCT complementation was superior to single-agent chemotherapy for the treatment of malignant lymphoma [12]. However, for HIV-positive patients, hematopoietic stem cells and progenitor cells have deficiencies with regard to erythropoiesis, myelopoiesis, and lymphopoiesis, and mobilization using G-CSF may be limited in specific cell populations. In addition, hematopoietic stem cells can be infected by HIV-1 and might become a latent reservoir of HIV [13]. Hence, the choice of ASCT or allogeneic hematopoietic cell transplantation is a major challenge. Considering the high risk of mortality and graft-versus-host-disease response in allogeneic hematopoietic cell transplantation, after obtaining written informed consent, we chose ASCT. We made this choice because there was no obvious bone marrow involvement after chemotherapy. The recovery time of neutrophils and platelets were 14 days and 25 days, respectively, which were similar to those in non-AIDS patients. In addition, we found after three cycles of chemotherapy, the EBV load was significantly reduced, without specific anti-EBV treatment; therefore, recovery of immunological function by cART and efficient chemoimmunotherapy might be associated with the reconstruction of the anti-EBV response. Although the sustained disappearance of EBV was regarded as a good prognostic marker for EBV-positive diffuse large B cell lymphoma [14], the reason for the good control of EBV in our patient requires further study.

Recently, two HIV patients with NHL at the time of the first remission and recurrence showed good tolerance to ASCT after a transplantation-conditioning regimen comprising a high dose of cyclophosphamide, carmustine, and etoposide [4]. Those results were confirmed by a large-scale study that suggested that patients with HIV should not be denied ASCT if they meet the standard criteria for transplantation [12]. However, until now, the efficacy of short-term high-intensity chemoimmunotherapy combined with ASCT has not been identified in patients with naïve or high-risk BL when accepting concomitant cART.

Here, we reported an HIV patient with naïve and high-risk BL who accepted short-term, high-dose chemotherapy followed by ASCT. cART was not stopped because of chemotherapy-related cytotoxicity, viral resistance did not occur, and complications were well controlled by effective symptomatic treatment. Given the poor outcome of HIV-related initial and high-risk Burkitt’s lymphoma, combined conventional chemotherapy with ASCT should be considered.

## Abbreviations

Ara-C: Cytosine arabinoside
ART: Antiretroviral therapy
ASCT: Autologous stem cell transfusion
BL: Burkitt’s lymphoma
cART: Combined antiretroviral therapy
FDG: Fludeoxyglucose
G-CSF: Granulocyte-colony stimulating factor
HIV: Human immunodeficiency virus
HRLs: HIV-associated lymphomas
HSCT: Hematopoietic stem cell transplantation
LDH: Lactate dehydrogenase
PBSCs: Peripheral blood stem cells
PET–CT: Positron emission tomography–computed tomography
RBCs: Red blood cells
WBC: White blood cell
